# How organizational commitment shapes IPC execution through job satisfaction among infection prevention and control professionals

**DOI:** 10.3389/fpubh.2026.1899025

**Published:** 2026-08-21

**Authors:** Lu Wang, Qianning Wang, Zhen Qin, Feiyang Zheng, Xinping Zhang, Tiantian Yu

**Affiliations:** 1 Department of Nursing, Hubei University of Traditional Chinese Medicine, Wuhan, Hubei, China; 2 Department of Global Health, School of Public Health, Peking University, Beijing, China; 3 Department of Epidemiology and Biostatistics, School of Public Health, Peking University, Beijing, China; 4 School of Medical Humanities and Management, Hangzhou Medical College, Zhejiang, China; 5 Department of Medicine and Health Management, Huazhong University of Science and Technology, Wuhan, Hubei, China

**Keywords:** infection prevention and control, IPC professionals, IPC SR-execution, job satisfaction, mediating effect, organizational commitment

## Abstract

**Background:**

Healthcare-associated infections underscore the critical role of infection prevention and control (IPC), a concern sharpened by the COVID-19 pandemic. Although organizational commitment and job satisfaction are recognized drivers of IPC compliance, their combined association with IPC execution and the mechanism linking them remain insufficiently understood. This study quantitatively examined the association between organizational commitment and IPC self-reported execution (SR-execution), with job satisfaction as a mediator.

**Methods:**

A cross-sectional study was conducted among 394 IPC professionals from 231 hospitals in Hubei Province, China. Organizational commitment comprised value, effort, and retention commitment (Cronbach’s *α* = 0.994); job satisfaction comprised coworker relationships, professional opportunities, praise and recognition, and extrinsic rewards (Cronbach’s *α* = 0.971); and IPC self-reported execution (SR-execution) comprised motivation, process, and outcomes (Cronbach’s α = 0.975). All items were rated on a five-point Likert scale. Structural equation modeling (SEM) was used to assess the proposed mediation framework.

**Results:**

The SEM demonstrated acceptable fit (χ^2^/df = 3.482, RMSEA = 0.079, CFI = 0.918, TLI = 0.906, IFI = 0.918, PGFI = 0.666). Organizational commitment was positively associated with job satisfaction (*β* = 0.628, *p* < 0.001) and IPC SR-execution (*β* = 0.556, *p* < 0.001). Job satisfaction was positively associated with IPC SR-execution (*β* = 0.307, *p* < 0.001). Job satisfaction significantly mediated the association between organizational commitment and IPC SR-execution (*β* = 0.193, SE = 0.052, 95% CI = 0.094–0.295), accounting for 25.78% of the total effect.

**Conclusion:**

Organizational commitment was positively associated with IPC SR-execution, and job satisfaction was a key mediator. These findings identify modifiable organizational factors that may strengthen IPC professionals’ implementation of IPC practices.

## 1 Introduction

Infection prevention and control (IPC) is fundamental to reducing healthcare-associated infections (HAIs) and protecting patients and healthcare workers. Effective IPC depends not only on guidelines and resources but also on the consistent implementation of everyday practices, including hand hygiene, appropriate use of personal protective equipment, environmental cleaning, and surveillance. Although these measures received heightened attention during the COVID-19 pandemic, sustaining high-quality IPC execution remains challenging (1–8).

Improving healthcare workers’ knowledge, attitudes, and practices (KAP) is central to HAI prevention. Evidence from Lebanon and Ethiopia shows that knowledge alone does not reliably translate into safe practice; continuing education, supportive supervision, and an enabling work environment are needed to convert knowledge and positive attitudes into consistent IPC behaviors (9, 10). Accordingly, IPC execution should be understood as both a technical and an organizational behavior.

The COVID-19 pandemic also intensified workload, occupational health risks, and psychological strain among healthcare workers. When high work effort is not matched by adequate recognition, remuneration, or organizational support, effort–reward imbalance may contribute to burnout and weaker adherence to standard precautions (11). Likewise, research among nursing students in Saudi Arabia found incomplete compliance with standard precautions, underscoring the importance of a supportive learning and clinical environment that reinforces safe practice (12). These findings suggest that the conditions in which healthcare workers perform IPC are as important as their technical knowledge.

**Figure 1:**
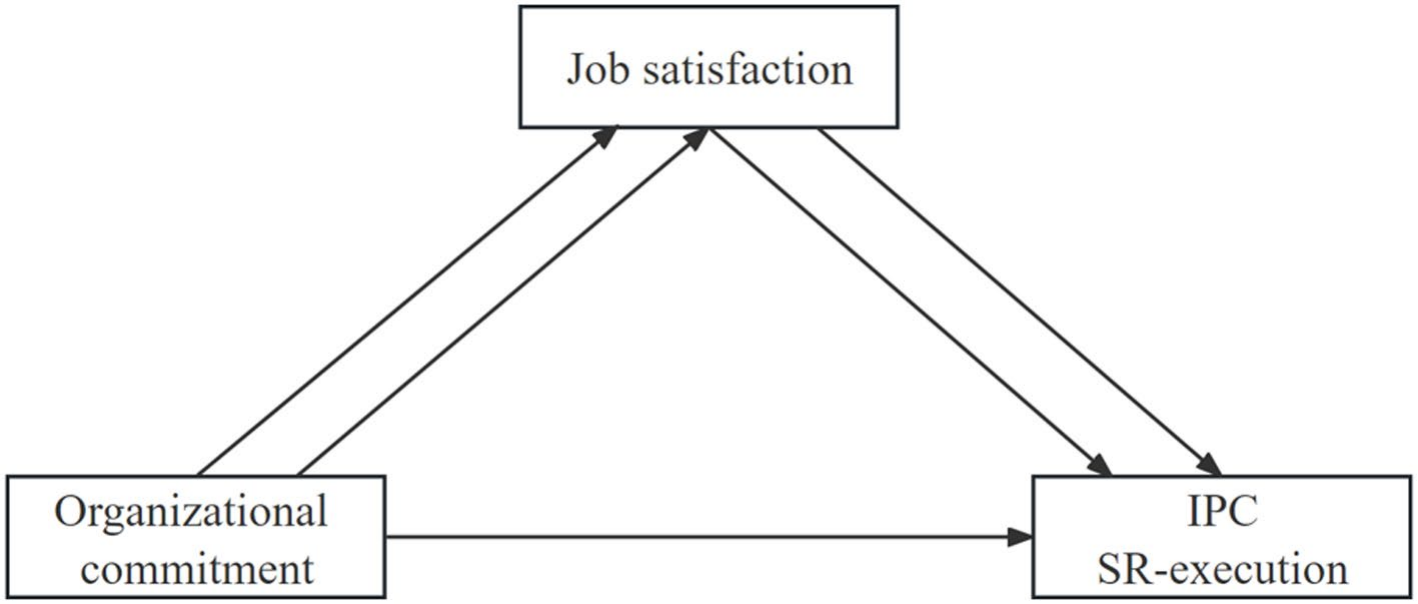
Conceptual framework of organizational commitment, job satisfaction, and IPC SR-execution.

Organizational commitment reflects employees’ identification with and willingness to contribute to their organization, whereas job satisfaction captures their appraisal of work, recognition, relationships, and rewards. Both factors may influence IPC SR-execution by shaping motivation, engagement, and adherence to organizational goals (Figure 1). However, their integrated association with IPC SR-execution has received limited quantitative attention. Therefore, this study aimed to examine the associations of organizational commitment and job satisfaction with IPC SR-execution among IPC professionals and to assess whether job satisfaction mediates the association between organizational commitment and IPC SR-execution.

## 2 Methods

### 2.1 Study design, participants, and quality control

#### 2.1.1 Study design

A cross-sectional study was conducted using an online self-administered questionnaire in hospitals across Hubei Province, Central China, from December 2024 to May 2025. A total of 231 hospitals were surveyed, accounting for 75.24% of all hospitals in the province.

#### 2.1.2 Participants and sample size

Participants were IPC professionals, defined as healthcare staff working in the Hospital-Acquired Infection Control Departments of the surveyed hospitals. The inclusion criteria were that IPC professionals were on duty and willing to complete the survey. A total of 419 responses were collected, of which 394 were valid, yielding an effective response rate of 94.03%.

#### 2.1.3 Quality control

The Quality Control Center for Hospital Infection Management in Hubei Province oversaw the administration of the survey. The center shared the questionnaire link with the directors of Hospital-Acquired Infection Control Departments in the participating hospitals. These directors then distributed the link to their departmental staff. To ensure the accuracy and quality of responses, IPC leaders reminded participants to complete the questionnaire diligently and provide precise information. Additional quality control measures were implemented before data analysis, based on the following criteria:

1. IPC professionals included in the analysis must have had at least one year of work experience.
2. The time taken to complete the questionnaire was required to be at least 10 min, based on a minimum completion time of 12 min determined in pre-testing.

### 2.2 Measurements

The questionnaire assessed organizational commitment, job satisfaction, IPC SR-execution, and participant demographics, including age and professional title. It was developed specifically for this study and is provided in the Supplementary material.

#### 2.2.1 Organizational commitment

Organizational commitment was assessed using a modified three-dimensional scale from Chang’s study, comprising value commitment, effort commitment, and retention commitment (8 items) (13). To suit the IPC context, statements were adjusted, such as modifying “I pay attention to the future development of the hospital” to “I pay attention to the future development of the department”. After modification, the scale exhibited excellent internal consistency (Cronbach’s *α* = 0.994) and acceptable construct validity, with factor loadings ≥ 0.61.

#### 2.2.2 Job satisfaction

Job satisfaction was measured using an adapted version of the MMSS scale, covering coworker relationships, professional opportunities, praise and recognition, and extrinsic rewards (8 items) (14). Adjustments were made to align with the IPC context, such as revising “Encourage or feedback” to “Identifying hidden dangers or unsafe IPC practices will be encouraged and receive feedback.” The revised scale demonstrated strong internal consistency (Cronbach’s *α* = 0.971) and acceptable construct validity, with factor loadings ≥ 0.66.

#### 2.2.3 IPC SR-execution

IPC SR-execution was measured using a modified three-dimensional scale from Wang’s study, encompassing SR-execution motivation, SR-execution process, and SR-execution outcomes (8 items) (6, 7). Examples include, “I execute IPC work effectively” categorized under the SR-execution outcome dimension. The scale demonstrated strong internal consistency (Cronbach’s *α* = 0.975) and acceptable construct validity, with factor loadings ≥ 0.71.

#### 2.2.4 Response format

All items were rated on a five-point Likert scale, where 1 indicated “strongly disagree” and 5 indicated “strongly agree.” Higher scores reflected higher levels of organizational commitment, job satisfaction, and IPC SR-execution.

### 2.3 Statistical analysis

Data were analyzed using SPSS (version 19.0, IBM Corp., Armonk, NY, USA) and AMOS (version 22.0, IBM Corp., Armonk, NY, USA) software.

#### 2.3.1 Descriptive statistics

Demographic characteristics of the participants were summarized using descriptive statistics, with continuous variables presented as mean ± standard deviation (Mean ± SD) and categorical variables as percentages (%).

#### 2.3.2 Correlation analysis

Pearson’s correlation coefficients were calculated to conduct a preliminary analysis of the associations among organizational commitment, job satisfaction, and IPC SR-execution.

#### 2.3.3 Structural equation modeling (SEM)

Associations among organizational commitment, job satisfaction, and IPC SR-execution were examined using SEM with maximum likelihood estimation. The analysis estimated direct effects and the indirect effect of job satisfaction in the association between organizational commitment and IPC SR-execution. The mediation effect was evaluated using bootstrap confidence intervals.

#### 2.3.4 Model fit evaluation

The goodness-of-fit of the SEM model was assessed using standard indices: χ^2^/df < 5, Root Mean Square Error of Approximation (RMSEA) < 0.08, Comparative Fit Index (CFI) > 0.90, Tucker-Lewis Index (TLI) > 0.90, Incremental Fit Index (IFI) > 0.90, Parsimony-Adjusted Goodness-of-Fit Index (PGFI) > 0.50.

## 3 Results

### 3.1 Demographic characteristics of IPC professionals

As shown in Table 1, the mean age of the participants was 40.53 years (SD = 8.86, range: 21–64). The average years of professional experience was 18.52 (SD = 10.13), indicating that the sample generally had substantial work experience. The majority of the participants were female (80.46%), while males accounted for 19.54%, reflecting the female-dominated nature of the IPC workforce.

**Table 1:** Demographic characteristics of IPC professionals (*N* = 394).

Characteristics	Mean ± SD/*N* (%)
Age (in years)	40.53 ± 8.86
Years of professional experience	18.52 ± 10.13
Gender	
Male	77 (19.54)
Female	317 (80.46)
Professional title	
None	8 (2.03)
Junior	96 (24.37)
Middle	175 (44.42)
Sub-senior	96 (24.36)
Senior	19 (4.82)
Highest level of education	
Associate Degree	78 (19.80)
Bachelor’s Degree	277 (70.30)
Master’s Degree	35 (8.88)
Doctorate	4 (1.02)

In terms of professional title, most participants held middle-level positions (44.42%), followed by junior (24.37%) and sub-senior titles (24.36%). Only a small proportion held senior titles (4.82%) or had no professional title (2.03%), suggesting that the sample was primarily composed of mid-level and above professionals.

Regarding educational attainment, most participants held a bachelor’s degree (70.30%), while 19.80% had an associate degree and 8.88% had a master’s degree or higher. Overall, the sample was characterized by a relatively well-educated and experienced group of IPC professionals.

### 3.2 Descriptive and correlation analysis among organizational commitment, job satisfaction, and IPC SR-execution

The mean scores for organizational commitment, job satisfaction, and IPC SR-execution ranged from 4.290 to 4.530, indicating relatively high levels across these variables. Specifically, organizational commitment had the highest mean score (4.530 ± 0.538), followed by IPC SR-execution (4.498 ± 0.491), while job satisfaction showed a comparatively lower, yet still high, mean score (4.290 ± 0.627). The relatively small standard deviations suggest a moderate level of variability among participants, indicating that most respondents reported consistently high perceptions across these constructs.

Correlation analysis revealed that organizational commitment was positively associated with both job satisfaction (r = 0.573, *p* < 0.01) and IPC SR-execution (r = 0.727, *p* < 0.01). The correlation between organizational commitment and IPC SR-execution was particularly strong, suggesting that higher levels of commitment are closely linked to better self-reported IPC execution. Additionally, job satisfaction was positively correlated with IPC SR-execution (r = 0.622, *p* < 0.01), indicating that individuals with greater satisfaction tend to report higher levels of IPC-related performance.

Overall, all key variables were significantly and positively correlated at the 0.01 level, providing preliminary support for the proposed relationships among organizational commitment, job satisfaction, and IPC SR-execution. The results are presented in Table 2.

**Table 2:** Descriptive and correlation analysis among organizational commitment, job satisfaction, and IPC SR-execution.

Variable	Mean	SD	Correlation coefficient		
			Organizational commitment	Job satisfaction	IPC SR-execution
Organizational commitment	4.530	0.538	1		
Job satisfaction	4.290	0.627	0.573**	1	
IPC SR-execution	4.498	0.491	0.727**	0.622**	1

**p < 0.01.

### 3.3 Total, direct, and indirect effects of organizational commitment on IPC SR-execution mediated by job satisfaction

**Figure 2:**
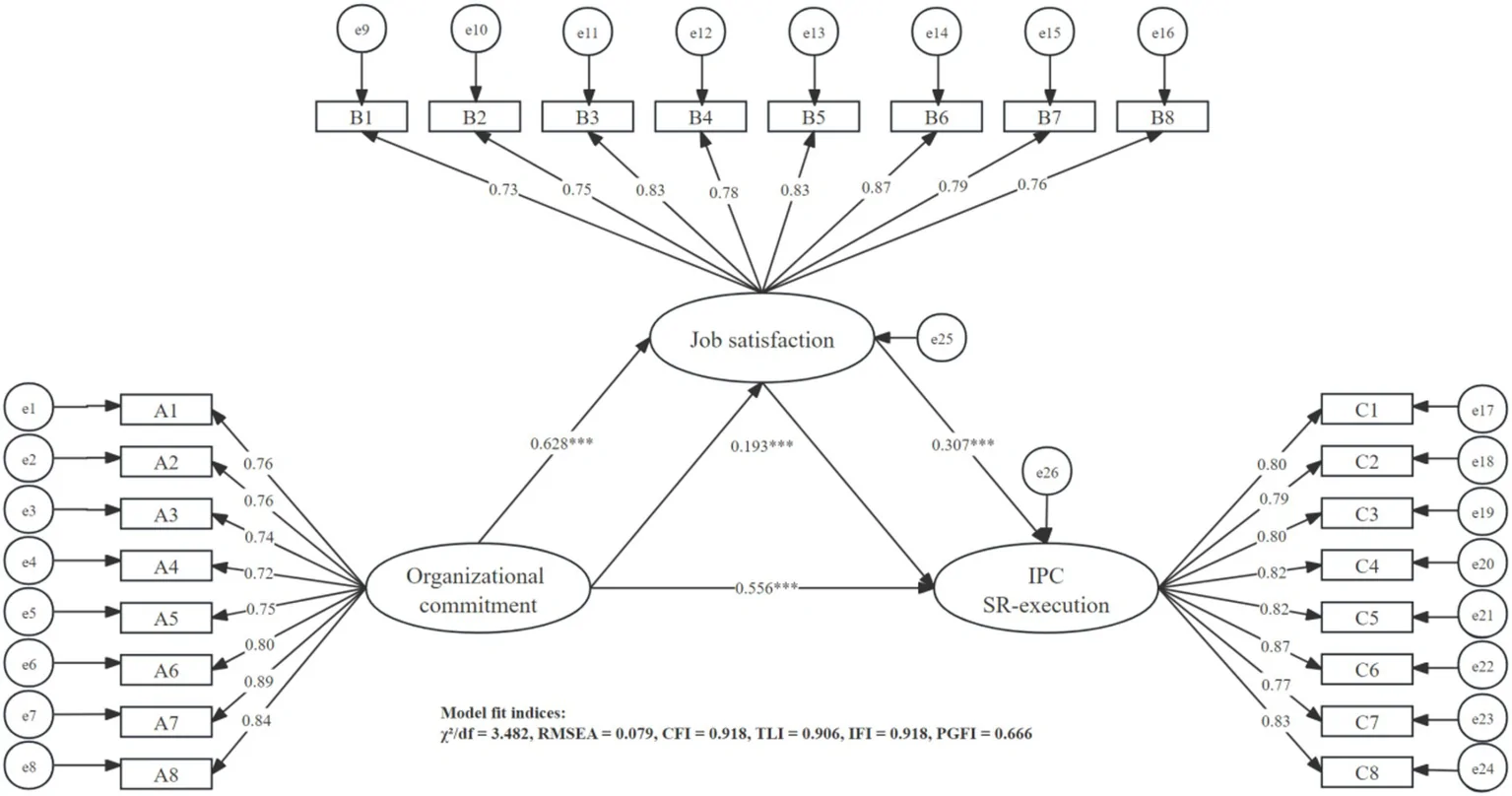
Structural equation model of organizational commitment, job satisfaction, and IPC SR-execution. A1-A8 refer to the items of organizational commitment, B1-B8 refer to the items of job satisfaction, and C1-C8 refer to the items of IPC SR-execution; ***p < 0.001.

The SEM analysis indicated acceptable fit for the proposed framework (χ^2^/df = 3.482, RMSEA = 0.079, CFI = 0.918, TLI = 0.906, IFI = 0.918, PGFI = 0.666), suggesting that the model adequately represented the relationships among the variables. Figure 2 presents the standardized path coefficients; all estimated paths were statistically significant (*p* < 0.001).

As shown in Table 3, organizational commitment exerted both direct and indirect effects on IPC SR-execution through job satisfaction. Specifically, organizational commitment had a significant positive effect on job satisfaction (*β* = 0.628, *p* < 0.001), indicating that higher levels of commitment were associated with greater satisfaction among IPC professionals. In addition, organizational commitment directly influenced IPC SR-execution (*β* = 0.556, *p* < 0.001), demonstrating its strong and independent contribution to execution outcomes.

**Table 3:** Total, direct, and indirect effects of organizational commitment on IPC SR-execution mediated by job satisfaction.

Path	Estimate	SE	*p*	95% CI	
				Lower	Upper
Total effect	0.749	0.081	< 0.001	0.595	0.908
Direct effect (X → M)	0.628	0.076	< 0.001	0.479	0.777
Direct effect (M → Y)	0.307	0.074	< 0.001	0.162	0.441
Direct effect (X → Y)	0.556	0.071	< 0.001	0.417	0.695
Indirect effect (X → M → Y)	0.193	0.052	< 0.001	0.094	0.295

X refers to organizational commitment, M refers to job satisfaction, Y refers to IPC SR-execution.

Job satisfaction, in turn, had a significant positive effect on IPC SR-execution (*β* = 0.307, *p* < 0.001), suggesting that more satisfied professionals were more likely to report better execution of IPC practices. The total effect of organizational commitment on IPC SR-execution was 0.749 (SE = 0.081, 95% CI = [0.595, 0.908]), reflecting a substantial overall influence.

Importantly, the indirect effect of organizational commitment on IPC SR-execution via job satisfaction was also statistically significant (*β* = 0.193, SE = 0.052, 95% CI = [0.094, 0.295]), accounting for 25.78% of the total effect. This indicates that job satisfaction partially mediates the relationship between organizational commitment and IPC SR-execution. In other words, while organizational commitment directly enhances IPC execution, it also exerts an additional influence by improving job satisfaction, which in turn promotes better execution outcomes.

Overall, these findings provide strong empirical support for the proposed mediation model and highlight the dual pathways, both direct and indirect, through which organizational commitment influences IPC SR-execution.

## 4 Discussion

This study explored the factors influencing IPC SR-execution from an organizational psychological perspective and examined the pathways through which organizational commitment is associated with IPC SR-execution. Notably, it represents one of the first attempts to investigate the mediating role of job satisfaction in this relationship using SEM, thereby providing a more integrative understanding of how organizational and psychological factors jointly shape IPC practices. By incorporating both direct and indirect pathways into a unified analytical framework, this study advances existing research that has traditionally examined these variables in isolation.

Our findings demonstrated that IPC professionals with higher levels of organizational commitment reported significantly greater job satisfaction. The relationship between organizational commitment and job satisfaction has been widely discussed in the literature, with consistent evidence supporting a positive association. Mosadeghrad suggested that organizational commitment serves as a key determinant of employee satisfaction, emphasizing its causal influence (15). Similarly, empirical research conducted in China found that organizational commitment was a significant predictor of nurses’ job satisfaction (*p* < 0.05), further reinforcing this link (16). The present study extends these findings to IPC professionals, a group that has received relatively limited attention in organizational behavior research, particularly in the context of infection prevention and control. From a theoretical perspective, this relationship can be explained by social exchange theory and organizational support theory, which posit that when employees perceive alignment with organizational values and feel supported by their institutions, they are more likely to develop positive attitudes toward their work, including higher satisfaction.

In addition to confirming this relationship, our study found that job satisfaction directly and positively influences IPC SR-execution. This finding is consistent with a large body of research in healthcare management, which highlights job satisfaction as a critical determinant of employee performance and behavioral compliance. In clinical settings, satisfied healthcare workers are more likely to be engaged, attentive, and motivated to adhere to established protocols, thereby improving both care processes and outcomes (17, 18). For example, higher job satisfaction among critical care nurses has been associated with increased adherence to central line bundle interventions and a reduction in central line-associated bloodstream infections (*p* < 0.05) (17). Conversely, low job satisfaction has been linked to higher rates of healthcare-associated infections, potentially due to decreased motivation, emotional exhaustion, and negative coping behaviors, such as noncompliance with IPC guidelines (18). These findings underscore the importance of maintaining high levels of job satisfaction among IPC professionals to ensure consistent and effective implementation of infection control measures.

Furthermore, this study confirmed that organizational commitment has a direct and significant positive effect on IPC SR-execution, independent of job satisfaction. This suggests that organizational commitment not only enhances employees’ attitudes but also directly influences their behaviors. One possible explanation is that organizational commitment fosters a sense of responsibility and accountability, motivating employees to go beyond minimum job requirements and actively engage in IPC practices (19). Previous studies have similarly shown that organizational commitment is associated with higher levels of compliance with safety protocols and organizational policies (7). For instance, research on infection control practices has demonstrated that organizational commitment, along with self-expectation leadership, significantly influences healthcare workers’ adherence to IPC measures (7). Additionally, strengthening organizational commitment has been shown to improve the safety climate within healthcare settings, which in turn enhances compliance with personal protective equipment protocols (*p* < 0.05) (20). These findings align with our results and highlight the critical role of organizational commitment as both a motivational and behavioral driver in IPC management.

A key contribution of this study lies in its identification of job satisfaction as a significant mediator in the relationship between organizational commitment and IPC SR-execution. While previous studies have predominantly focused on the direct effects of organizational commitment or job satisfaction, our findings demonstrate that job satisfaction partially mediates this relationship, accounting for a substantial proportion of the total effect. This suggests that organizational commitment influences IPC SR-execution not only directly but also indirectly by enhancing employees’ satisfaction with their work. Such a mediating mechanism is consistent with findings from business management and organizational psychology, where job satisfaction has been shown to mediate the relationship between organizational factors and employee outcomes (21, 22). For example, job satisfaction has been found to partially mediate the relationship between job identity differences and employee creativity (*p* < 0.001) (22). By extending this line of research to the IPC context, our study provides new insights into the underlying mechanisms through which organizational factors shape infection control behaviors.

Importantly, although IPC professionals in this study reported relatively high levels of job satisfaction and organizational commitment, there remains room for further improvement (23). In particular, dimensions such as coworker relationships, professional development opportunities, recognition, and extrinsic rewards warrant greater attention (24). Enhancing these factors may not only improve job satisfaction but also strengthen organizational commitment, thereby creating a positive feedback loop that ultimately enhances IPC SR-execution. From a practical perspective, hospital managers and policymakers should prioritize interventions aimed at improving the work environment of IPC professionals. This may include providing more opportunities for professional growth, implementing fair and transparent reward systems, and fostering a culture of recognition and appreciation.

In addition, cultivating a supportive organizational culture and strengthening team dynamics are essential for improving both organizational commitment and job satisfaction. A collaborative environment characterized by trust, communication, and shared goals can enhance employees’ sense of belonging and engagement, which in turn promotes adherence to IPC protocols. Leadership also plays a crucial role in this process. Transformational leadership, in particular, has been shown to positively influence both organizational commitment and job satisfaction by inspiring employees, recognizing their contributions, and providing a clear vision for organizational goals. By integrating leadership development into IPC management strategies, healthcare institutions can further enhance the effectiveness of their infection control programs.

Another important implication of this study is the need to adopt a more holistic approach to IPC management. While traditional strategies have focused primarily on technical training, resource allocation, and policy enforcement, our findings suggest that psychological and organizational factors are equally important. Interventions that target only structural aspects may be insufficient if employees lack motivation or satisfaction. Therefore, integrating organizational behavior principles into IPC management may lead to more sustainable improvements in compliance and execution. For example, combining training programs with initiatives aimed at enhancing organizational commitment and job satisfaction could produce synergistic effects, leading to better outcomes than either approach alone.

Despite its contributions, this study has several limitations that should be acknowledged. First, as a cross-sectional study, causal relationships among organizational commitment, job satisfaction, and IPC SR-execution cannot be definitively established. Although SEM provides a robust framework for testing theoretical models, longitudinal or experimental studies are needed to confirm the directionality of these relationships. Second, this study did not include other potential predictors of IPC SR-execution, such as leadership style, organizational culture, workload, or individual psychological traits. Future research should incorporate these variables to develop a more comprehensive model. Third, the study sample was limited to IPC professionals in Hubei Province, which may restrict the generalizability of the findings to other regions or healthcare systems. Differences in organizational structures, cultural contexts, and resource availability may influence the observed relationships.

Future research could address these limitations by employing longitudinal designs, expanding the range of variables examined, and including more diverse samples across different regions and healthcare settings. Additionally, qualitative studies may provide deeper insights into the experiences and perceptions of IPC professionals, complementing the quantitative findings of this study.

## 5 Conclusion

Organizational commitment was positively associated with IPC SR-execution, and job satisfaction significantly mediated this association. These findings support organizational interventions that strengthen IPC professionals’ job satisfaction, recognition, professional development, and commitment, alongside ongoing IPC training and adequate resources.

## Data Availability

The raw data supporting the conclusions of this article will be made available by the authors, without undue reservation.
