# Supplementary material for: How organizational commitment shapes IPC execution through job satisfaction among infection prevention and control professionals
Source: Front Public Health. 2026 Aug 21;14:1899025. doi: 10.3389/fpubh.2026.1899025 (PMC13541698; doi:10.3389/fpubh.2026.1899025)
Supplement: Supplementary file 1 [file Supplementary_file_1.docx]

# Appendix 1 Questionnaire on the Influence of Health Opinion Leaders on Health-Promoting Behaviors among Rural Residents

Dear Respondent,

Hello! This questionnaire focuses on health opinion leaders and their influence on rural residents. The survey is solely for academic research purposes. Your anonymity is guaranteed, and there are no right or wrong answers. Please answer according to your actual situation.

A **health opinion leader** refers to an individual who, by virtue of their own qualities and capabilities—such as rich health information, professional practical skills, or broad communication channels—influences residents' health attitudes and behaviors. Examples include doctors, fitness enthusiasts, medical hobbyists, and internet influencers/bloggers. The term "opinion leader" used briefly in the questionnaire refers specifically to health opinion leaders.

Tongji Medical College, Huazhong University of Science and Technology

**For the following statements, please rate the overall level of your health opinion leaders.**

| **Part 1: Influence of Health Opinion Leaders on Health-Promoting Behaviors among Rural Residents** | | | | | |
| --- | --- | --- | --- | --- | --- |
| **In your daily life, regarding health-related aspects (such as diet, exercise, mental health, interpersonal relationships, seeking medical care, etc.), whose advice do you follow the most? (Multiple choice)**  □1. Village cadres □2. Educated young people □3. Family members □4. Medical hobbyists  □5. Migrant workers / successful businesspeople □6. Doctors / Nurses □7. Respected elderly  □8. Team leaders or group leaders □9. Village enterprise / industry leaders  □10. Health bloggers / Internet influencers □11. Myself _________ (Please state your role/identity)  □12. Other _________ (Please specify) | | | | | |
| I. Expertise | Strongly Disagree | Disagree | Neutral | Agree | Strongly Agree |
| (1) Opinion leaders possess a deep understanding of health knowledge. | □ | □ | □ | □ | □ |
| (2) Opinion leaders master health skills (e.g., measuring temperature, blood pressure, blood sugar; rational medication use; CPR/first aid). | □ | □ | □ | □ | □ |
| (3) Opinion leaders can solve or help me solve health problems (e.g., diet, medication usage, vaccination, seeking medical care). | □ | □ | □ | □ | □ |
| (4) Opinion leaders have practical health experience (e.g., frequent medical visits, long-term self-health/disease management, frequent participation in health activities). | □ | □ | □ | □ | □ |
| (5) Opinion leaders have conducted or provided me with health training or guidance (e.g., health education, lectures, science popularization). | □ | □ | □ | □ | □ |
| II. Communication Power | Strongly Disagree | Disagree | Neutral | Agree | Strongly Agree |
| (1) Opinion leaders use diverse methods to disseminate health information (e.g., face-to-face, online multimedia platforms, patient groups, WeChat groups, various graphic/text formats). | □ | □ | □ | □ | □ |
| (2) Opinion leaders disseminate rich and varied health information content (e.g., health topics, health articles, images, videos, recommendations for medicines/health products). | □ | □ | □ | □ | □ |
| (3) The health information disseminated by opinion leaders has broad coverage (e.g., high awareness, likes, shares, views, and topic engagement when forwarded in Moments/WeChat groups). | □ | □ | □ | □ | □ |
| (4) The health information disseminated by opinion leaders is recognized by me or others. | □ | □ | □ | □ | □ |
| (5) The health information disseminated by opinion leaders is correct and reliable. | □ | □ | □ | □ | □ |
| III. Organizational Ability | Strongly Disagree | Disagree | Neutral | Agree | Strongly Agree |
| (1) Opinion leaders organize and plan health-related activities (e.g., square dancing, badminton, walking, live exercise broadcasts, lectures). | □ | □ | □ | □ | □ |
| (2) Opinion leaders possess good communication and coordination skills when organizing health activities. | □ | □ | □ | □ | □ |
| (3) Opinion leaders can flexibly choose locations, methods, and handle unexpected situations during health-related activities based on actual circumstances. | □ | □ | □ | □ | □ |
| (4) Opinion leaders encourage me or others to participate in the health activities they organize. | □ | □ | □ | □ | □ |
| (5) The health activities organized by opinion leaders can be carried out smoothly. | □ | □ | □ | □ | □ |
| IV. Innovativeness | Strongly Disagree | Disagree | Neutral | Agree | Strongly Agree |
| (1) Opinion leaders have innovative and creative ideas about health (e.g., health runs, cultural/sports activities, environmental sanitation improvements, rural toilet upgrades). | □ | □ | □ | □ | □ |
| (2) Opinion leaders propose novel solutions to health problems. | □ | □ | □ | □ | □ |
| (3) Opinion leaders plan and execute innovative and creative health activities. | □ | □ | □ | □ | □ |
| (4) Opinion leaders take the lead in participating in innovative and creative health activities. | □ | □ | □ | □ | □ |
| V. Interactivity | Strongly Disagree | Disagree | Neutral | Agree | Strongly Agree |
| (1) Opinion leaders respond to health-related questions or topics raised by me or others. | □ | □ | □ | □ | □ |
| (2) I or others respond to health topics or activities initiated by opinion leaders. | □ | □ | □ | □ | □ |
| (3) Opinion leaders can respond quickly to health-related questions or topics raised by me or others. | □ | □ | □ | □ | □ |
| (4) I or others can participate effectively and deeply in health topics or activities initiated by opinion leaders (e.g., actively expressing opinions, thinking, discussing). | □ | □ | □ | □ | □ |
| VI. Popularity | Strongly Disagree | Disagree | Neutral | Agree | Strongly Agree |
| (1) Opinion leaders have a certain level of fame and prestige. | □ | □ | □ | □ | □ |
| (2) Opinion leaders are well-known figures. | □ | □ | □ | □ | □ |
| (3) Opinion leaders have a certain degree of influence and appeal. | □ | □ | □ | □ | □ |
| (4) Opinion leaders hold a certain leadership position (e.g., medical experts, village cadres, village committees, women's federations, internet influencers, or the most influential person in the family). | □ | □ | □ | □ | □ |
| VII. Trust | Strongly Disagree | Disagree | Neutral | Agree | Strongly Agree |
| (1) I believe the opinion leader is honest. | □ | □ | □ | □ | □ |
| (2) I believe the opinion leader is unbiased when sharing information. | □ | □ | □ | □ | □ |
| (3) I believe the opinion leader possesses rich knowledge and skills. | □ | □ | □ | □ | □ |
| (4) I believe the information released by the opinion leader is valuable. | □ | □ | □ | □ | □ |
| (5) I believe the opinion leader will do their best to help others. | □ | □ | □ | □ | □ |
| (6) I believe the opinion leader considers others as well as themselves. | □ | □ | □ | □ | □ |
| VIII. Health Attitudes | Strongly Disagree | Disagree | Neutral | Agree | Strongly Agree |
| (1) Learning about health and disease knowledge is important to me. | □ | □ | □ | □ | □ |
| (2) Good diet and lifestyle habits are beneficial to me. | □ | □ | □ | □ | □ |
| (3) Regular check-ups can prevent diseases. | □ | □ | □ | □ | □ |
| (4) Health education is beneficial to me. | □ | □ | □ | □ | □ |
| (5) Physical exercise can improve health. | □ | □ | □ | □ | □ |
| (6) Frequently experiencing negative emotions (anxiety, depression, etc.) is harmful to health. | □ | □ | □ | □ | □ |
| IX. Health-Promoting Behaviors | | Never | Occasionally | Often | Always |
| (1) I eat bread, rice, noodles/pasta, and cereal foods daily. | | □ | □ | □ | □ |
| (2) I eat fruit daily. | | □ | □ | □ | □ |
| (3) I eat vegetables daily. | | □ | □ | □ | □ |
| (4) I eat meat, poultry, fish, dried beans, eggs, and nuts daily. | | □ | □ | □ | □ |
| (5) I read food labels (e.g., expiration date, storage instructions). | | □ | □ | □ | □ |
| (6) I eat breakfast daily. | | □ | □ | □ | □ |
| (7) I follow a planned exercise routine. | | □ | □ | □ | □ |
| (8) I engage in high-intensity exercise at least three times a week (e.g., brisk walking, cycling, stair climbing, lion/dragon dances, stilt walking, yangge dance, singing traditional opera; ≥20 min/session). | | □ | □ | □ | □ |
| (9) I engage in light to moderate physical activity weekly (e.g., walking 30-40 min, gardening/yard work, yoga, Tai Chi). | | □ | □ | □ | □ |
| (10) I participate in recreational and leisure activities (e.g., playing cards, dancing). | | □ | □ | □ | □ |
| (11) I do stretching exercises at least three times a week (e.g., bending, kicking, chest expansion exercises). | | □ | □ | □ | □ |
| (12) I incorporate exercise into my daily life (e.g., walking after meals, walking instead of taking transport when possible). | | □ | □ | □ | □ |
| (13) I measure my pulse during exercise. | | □ | □ | □ | □ |
| (14) I reach my target heart rate during exercise. | | □ | □ | □ | □ |
| (15) I am actively growing and changing. | | □ | □ | □ | □ |
| (16) My life has purpose. | | □ | □ | □ | □ |
| (17) I look forward to the future. | | □ | □ | □ | □ |
| (18) I work towards my long-term life goals. | | □ | □ | □ | □ |
| (19) I know what is important in life. | | □ | □ | □ | □ |
| (20) I am happy to praise others' successes. | | □ | □ | □ | □ |
| (21) I maintain meaningful interpersonal relationships. | | □ | □ | □ | □ |
| (22) I enjoy being with good friends. | | □ | □ | □ | □ |
| (23) I voluntarily offer care, love, and warmth to others. | | □ | □ | □ | □ |
| (24) I receive social support from my interpersonal network. | | □ | □ | □ | □ |
| (25) I choose foods low in fat, saturated fat, and cholesterol (e.g., eat less fried food, prefer vegetable oils). | | □ | □ | □ | □ |
| (26) I limit my intake of sugar and sugary foods. | | □ | □ | □ | □ |
| (27) I perform self-examinations of my body at least once a month. | | □ | □ | □ | □ |
| (28) I read or watch health promotion materials, videos, magazines, or TV programs. | | □ | □ | □ | □ |
| (29) I participate in health education activities. | | □ | □ | □ | □ |
| (30) I discuss health issues with health professionals. | | □ | □ | □ | □ |
| (31) I consult health professionals on how to maintain health. | | □ | □ | □ | □ |
| (32) I ask questions to health professionals and understand their guidance. | | □ | □ | □ | □ |
| (33) I consult health professionals about any abnormal symptoms or signs I have. | | □ | □ | □ | □ |
| (34) When I distrust the advice of a health professional, I seek a second opinion. | | □ | □ | □ | □ |
| (35) I seek guidance and counseling when necessary. | | □ | □ | □ | □ |
| (36) I find some time to relax myself every day. | | □ | □ | □ | □ |
| (37) I accept things in life that I cannot change. | | □ | □ | □ | □ |
| (38) I think of happy things before sleeping. | | □ | □ | □ | □ |
| (39) I take measures to relieve stress. | | □ | □ | □ | □ |
| (40) I maintain peace of mind and avoid fatigue. | | □ | □ | □ | □ |
| **Part 2: Basic Information** | | | | | |
| (1) Gender: □ Male □ Female | | | | | |
| (2) Age: _________ years old | | | | | |
| (3) Education Level: □ Primary school or below □ Junior high school □ High school / Technical secondary school □ College □ Bachelor's degree or above | | | | | |
| (4) Health Status: □ Poor □ Fair □ Good □ Excellent | | | | | |
| (5) Average Annual Income (10,000 RMB): □ ≤1 □ 1-3 □ 3-5 □ 5-8 □ ≥8 | | | | | |
| (6) Marital Status: □ Other □ Married | | | | | |
| (7) Number of close relationships: □ None □ Few □ Moderate □ Many | | | | | |
| (8) In the past year, I have received health education (e.g., browsing health materials, attending health lectures, counseling activities, physical examinations): □ No □ Yes | | | | | |
| (9) I have the ability to manage my own health: □ Strongly disagree □ Disagree □ Neutral □ Agree □ Strongly agree | | | | | |
| (10) My level of health knowledge mastery: □ Completely unaware □ Know a little □ Know some □ Know most □ Know everything | | | | | |
